# Yoga as a method of symptom management in multiple sclerosis

**DOI:** 10.3389/fnins.2015.00133

**Published:** 2015-04-30

**Authors:** Rachael Frank, Jennifer Larimore

**Affiliations:** Department of Biology and Neuroscience Program, Agnes Scott CollegeDecatur, GA, USA

**Keywords:** multiple sclerosis, yoga, intervention, spasticity, mobility, mental health

## Abstract

Multiple Sclerosis (MS) is an immune-mediated process in which the body's immune system damages myelin in the central nervous system (CNS). The onset of this disorder typically occurs in young adults, and it is more common among women. Currently, there is no cure and the long-term disease progression makes symptomatic management critical for maintaining quality of life. Several pharmacotherapeutic agents are approved for treatment, but many patients seek complementary and alternative interventions. Reviews have been conducted regarding broad topics such as mindfulness-based interventions for people diagnosed with MS and the impact of yoga on a range of neurological disorders. The objective of the present review is to examine the potential benefits of yoga for individuals with MS and address its use in managing symptoms including pain, mental health, fatigue, spasticity, balance, bladder control, and sexual function.

## Introduction

Multiple Sclerosis (MS) was first recognized as a disease by the French neurologist Jean-Martin Charcot in 1868. MS is a chronic neurological disorder in which the body's immune system mounts an attack against the central nervous system (CNS). Within the CNS, oligodendrocytes produce the fatty substance myelin, which wraps neuronal axons and allows for effective transmission of signals. The T cell-mediated immune response associated with MS is directed toward oligodendrocytes and myelin, producing a devastating effect. Demyelination is responsible for impaired neuronal conduction and the symptoms seen in patients with the disease. In addition to the inflammatory effect on myelin, axonal injury occurs and is characterized by accumulation of amyloid precursor protein (Bitsch et al., 2000). Common symptoms are varied and may include fatigue, spasticity, impairment or loss of mobility, bladder and bowel dysfunction, chronic pain, depression, and cognitive impairment (Senders et al., 2012; Ahmadi et al., 2013).

The onset of the disease typically occurs in young adults ages 20–40, and is far more prevalent in females than in males. It is estimated that MS affects 400,000 people in the United States and more than 2.5 million people worldwide. There are four courses categorizing this degenerative disease, which were initially described in 1996 by the National Multiple Sclerosis Society in the US. An international panel later adopted these four courses in 2013. Of the four, relapse remitting (RRMS) is the most common diagnosis. Over time, this typically progresses into a secondary progressive (SPMS) course. The two less common forms of MS are primary progressive (PPMS) and progressive relapsing (PRMS) (Tullman, 2013). For patients with RRMS, onset of new symptoms occurs during periods of relapse, which are followed by remission periods of varying lengths. In the majority of these patients, RRMS develops into SPMS, and the disease progresses without periods of remission. Individuals diagnosed with PPMS experience increasing neurologic dysfunction without periods of remission from disease onset, and those with PRMS experience a steady decrease in neurologic function accompanied by occasional exacerbations and worsening of symptoms. MS is not considered fatal and most people have a near-normal life expectancy after diagnosis, although there is tremendous variability in the rate of progression between individuals.

Presently, there is no cure for MS. Therefore, the chronic nature of the disease makes managing disease progression and symptoms imperative for maintaining quality of life. There are currently 12 disease-modifying medications approved by the U.S. Food and Drug Administration (FDA) to reduce the severity of relapses and the accumulation of brain lesions, though approved MS medications may be expensive and insurance coverage varies widely. These medications are designed for patients with relapsing forms of MS, and no medications have been approved for treatment of PPMS. Relapses are commonly treated with corticosteroids, which have no long-term benefits, and there is a wide range of medications commonly used to manage ongoing symptoms. However, individuals may have adverse reactions to pharmacological treatments. Some medications may produce negative side effects such as nausea, fever, headache, fatigue, depression, and psychological imbalance (Petejan et al., 1996; Ahmadi et al., 2010). In response to the often-prohibitive costs of medications and the adverse side effects that may accompany certain drugs, patients with MS often use complementary and alternative treatments as methods of disease management. It is estimated that between 54 and 57% of patients in the United States with MS use some form of complementary or alternative treatment, and prevalence of these therapies may be even higher in other countries such as Great Britain (Hughes and Howard, 2013). Complementary and alternative medicine (CAM) includes a wide range of non-pharmacological interventions that are defined by the National Center for Complementary and Alternative Medicine (NCCAM) as “complementary” when used in addition to conventional medicine and “alternative” when used in place of it. Due to the complex progression of MS, The National Society for MS promotes a comprehensive approach for disease management, including exercise and complementary therapies such as yoga.

Exercise was once believed to increase the fatigue and troubles with becoming overheated that many MS patients experience, but is now acknowledged as an inexpensive and beneficial form of therapy (Ahmadi et al., 2013). An exercise regimen combining balancing, strengthening, stretching, and aerobic components can be beneficial and enjoyable for individuals with MS (Hale et al., 2003). Yoga is an ancient Indian practice that combines these elements with a philosophy of mind-body awareness. NCCAM defines yoga as a mind-body practice that incorporates breathing, physical postures, and relaxation. There are many different forms of yoga with varying intensities, crossover between practices, and diverse teaching styles. These practices include Hatha and Kundalini, which are gentler and focus on breath and poses; Iyengar, with a focus on poses and frequent use of props; Ashtanga and Vinyasa, typically more physically demanding; and Bikram yoga, taught in a heated setting (Senders et al., 2012). Some of these practices are more appropriate than others for people with MS, depending on the individual's physical abilities. Bikram yoga is likely to be hazardous for patients with MS due to the heat sensitivity associated with the disease, while practices incorporating props and cushions might be more appealing for patients with neurodegenerative disorders such as MS (Wahbeh et al., 2008).

Although effects on symptom management may vary between individuals, yoga's focus on breath, movement, and stretching may have therapeutic benefits for patients with MS and improve outcomes such as self-efficacy, mental health, and quality of life. Viewed in this context, yoga is a promising method of symptom management for patients with MS. Previous reviews have addressed impact of yoga on a range of neurological disorders (Mishra et al., 2012) and mindfulness-based interventions for MS (Senders et al., 2012). The objective of the present article is to examine the benefits of yoga for individuals with MS and address its use in managing symptoms including pain, mental health, fatigue, spasticity, balance, bladder control, and sexual function.

## Yoga as an intervention

Quality of life (QoL) is a broad concept, encompassing both physical and mental health components. Pain is one physical variable capable of affecting QoL, ability to work, and mental health. Pain is a commonly reported symptom for individuals with MS and manifestations include back pain, trigeminal neuralgia, painful spasms, extremity pain, and headache (O'Connor et al., 2008). Previous research has examined how pain and QoL were impacted by a 3-month intervention of pain-managing yoga techniques. In this clinical trial of 60 women with MS, the treatment group reported significantly lower pain (*p* = 0.007) and improved QoL (*p* = 0.001) following the intervention (Doulatabad et al., 2013). Based on this study, pain-managing yoga techniques enhanced QoL by decreasing pain in patience with MS.

The mental health component of QoL includes depression, cognition, stress, and mental fatigue. Depression, experienced by up to 50% of patients with MS, can impact cognition, willingness for treatment, and suicidal intent (Feinstein et al., 2013). Results of an 8-week yoga intervention targeting depression among women with MS, revealed significantly lower levels of depression in the experimental group following the treatment (*n* = 15, *p* < 0.05) and no change for the 15 participants in the control group (Rahnama et al., 2011). Another study examined the impact of different forms of exercise on cognition in 24 patients with RRMS, found participants showed significantly improved reaction time following treadmill walking, cycling, and yoga compared to resting (Sandroff et al., 2015). Walking corresponded to the sole significant reduction in the effect of interfering stimuli on reaction time (*p* = 0.04). However, participants engaged in only 20 min of each exercise, so these results may not be applicable to a longer exercise regime. In a study of a 6-week relaxation-based yoga intervention on a sample of chronically ill patients with cancer (*n* = 10) or MS (*n* = 12), both groups of patients reported significantly lower perceived stress (*p* < 0.001), with a greater effect for patients with MS (Pritchard et al., 2010). In additional studies, researchers examined the effect of an 8-week mindfulness-based intervention (MBI) using yoga on depression, anxiety, and fatigue in participants with RRMS or SPMS (Grossman et al., 2010). Participants who received the intervention (*n* = 76) showed improved levels of depression, anxiety, and fatigue (*p* < 0.001) compared to the control group (*n* = 74), and results remained significant at a 6-month follow up. This study employed an intensive intervention with weekly group meetings and “homework” for the participants to practice individually. Finally, in a 6-month randomized control trial investigating yoga's use in MS management, Oken et al. found that yoga improved levels of fatigue compared to the control (*N* = 57, *p* < 0.001), although the level of improvement was not significantly different than a conventional aerobic exercise regimen (Oken et al., 2004).

Other studies have yielded mixed results. In a 2013 study with 24 participants, self-reported measures following a 4-month yoga intervention indicated improvement in mental components of QoL (*p* = 0.01) and fatigue (*p* = 0.039), but physical components of QoL showed no significant change (Salgado et al., 2013). Participants in this study were in the early stages of MS and reported low levels of pain both before and after the intervention. In a separate study, a 10-week yoga intervention (Garrett et al., 2012a,b) had a similar outcome for the mental component of the study, but contrary results for the physical component. In this study, 63 participants with RRMS, SPMS, or PPMS and minimal gait impairment reported significant improvement compared to the control group in the psychological component of the MS Impact Scale-29 (*p* = 0.04), Modified Fatigue Impact Scale (MFIS) total (*p* = 0.05), and the MFIS physical subscale (*p* = 0.04). This intervention focused on relaxation, maintaining poses, and a series of joint freeing exercises to improve range of motion. Participants showed improvements in all categories except for gait during a 6-min walk test, although these results were not maintained at a follow-up 12 weeks later. After the intervention, participants no longer had access to the instructors, group exercises, and gym equipment used in the trial and cessation of exercise may have occurred. Finally, a study with participants between the ages 26–50, found a 17% increase in selective attention following the yoga intervention (*n* = 20, *p* = 0.015), although outcomes for mood and fatigue were not significant (Velikonja et al., 2010). Taken together, these studies indicate more research is necessary to understand the long-term effects of yoga for patients with MS.

Physical components of QoL that may be impacted by MS include spasticity, balance, strength, bladder function, and sexual function. Spasticity, prevalent in up to 80% of MS cases, may be manifested as muscle stiffness, muscle spasms, or cramping (Hughes and Howard, 2013). It may also negatively affect posture, gait, and mobility (Sosnoff and Motl, 2012). Veliknoja et al. found there was no significant improvement in spasticity following a yoga intervention for 20 patients with RRMS or progressive MS (Velikonja et al., 2010). However, because the trial was uncontrolled, more evidence is needed to examine the effects of yoga on spasticity. In a study conducted by Guner and Inanici, they found that a bi-weekly yoga program over the course of 12 weeks yielded significant improvements in balance (*p* = 0.027), fatigue (*p* = 0.012), step length (*p* = 0.007), and walking speed (*p* = 0.005) in a group of eight participants with MS (Guner and Inanici, 2015). Another study demonstrated a 4-month yoga intervention yielded improvements with moderate effect sizes in balance (*n* = 24, *p* = 0.002) and functional strength (*p* < 0.001) following the treatment (Salgado et al., 2013). A 2012 study tested the efficacy of yoga pelvic floor exercises and breathing techniques as a complementary method of treating Neurogenic Bladder Dysfunction (NBD) in 11 patients (Patil et al., 2012). Ultrasound scanning revealed significantly reduced post void residual urine volume (*p* = 0.004), frequency of micturition was reduced by 25% (*p* = 0.001), and improvements in self-reported measures of urogenital distress (*p* = 0.006) and incontinence impact (*p* = 0.003). This was not a randomized control trial, thus there was no control group. Impaired sexual function and decreased satisfaction may occur with MS. Additionally, certain pharmacological therapeutics, such as selective serotonin reuptake inhibitors, may include sexual dysfunction as a side effect (DeLuca and Nocentini, 2011). There is evidence suggesting that yoga may be an effective method for improving sexual function and physical activity among women with MS (Najafidoulatabad et al., 2014). A 3-month randomized control trial with a yoga intervention focusing on slow body movement, respiration, and concentration, reported participants who received the treatment reported improved levels of both physical activity and sexual satisfaction (*N* = 60, *p* = 0.001).

## Conclusion

Traditionally, healthcare providers advised patients with MS to limit physical activity in order to reduce levels of fatigue, although this attitude has shifted due to experimental results suggesting that exercise is beneficial for patients with MS and may not adversely affect fatigue (Smith et al., 2013). The literature suggests there are not only physical benefits of exercise but also benefits for mental health including neurogenesis, improved CNS metabolism, and improvements in learning and memory (Feinstein et al., 2013).

Also, beliefs about the role of CAM therapies and MBI in MS management have shifted (Simpson et al., 2014). Treatments such as yoga, cannabinoids, and acupuncture are becoming more widely endorsed (DeLuca and Nocentini, 2011). Yoga is regarded as safe, inexpensive, and may be more accessible for patients with spasticity and impaired mobility than other forms of exercise. A yoga intervention has been shown to be as beneficial as aerobic therapy (Oken et al., 2004), and may be more practical for some MS patients due to readily modifiable poses. In spite of evidence suggesting that individuals with MS may benefit from yoga as a complementary therapy, there are inconsistencies and limitations in experimental results.

A major limitation of studies on yoga and MS is that it is impossible to blind participants to the intervention, and since they are aware of the treatment, some may have an expectation of improvement, which could influence results (Senders et al., 2012). Additionally, it is difficult to discern whether improvement following interventions may be due to confounding variables. For instance, some benefits could be conferred through social interactions during group exercise (Bigi and Yeh, 2013) or the yoga instructor serving as an encouraging mentor (Kishiyama et al., 2002). In many studies, participants were assigned to either a treatment or control group, though evidence supporting the outcome is stronger in three-arm trials with an alternate exercise group that serves as an active control to the yoga intervention (Senders et al., 2012). Further limitations in previous studies are frequently small sample sizes and lack of random sampling.

Few studies have addressed the pathophysiology of exercise or yoga on spasticity in MS. Consequently, little is known about the possible affect of yoga on this symptom of the disease. Spasticity is partly due to stretch-induced stimulation in less cooperative muscles and changes in excitability of motor neurons and spinal interneurons, which could be impacted by exercise (Sosnoff and Motl, 2012). However, spasticity is rarely a primary outcome of interest in existing studies, although it may affect other variables such as gait and mobility.

There are inconsistencies in the impact of yoga on mental health. While some studies have shown yoga may reduce stress and improve mental health (Grossman et al., 2010; Pritchard et al., 2010), other studies have found no effect. There is cross-sectional data indicating that exercise is beneficial for cognitive function in individuals with MS, but there is a lack of random control trials with supporting evidence to strengthen these results (Motl et al., 2011). Depression may be difficult to study as a variable because some symptoms, such as mental fatigue and trouble sleeping, may present themselves as depression. Previous studies have not been rigorous in their methodology to study this outcome or included clinically depressed participants in their sample. Some studies may evaluate outcome on depressed mood, but not have thorough psychological assessment of depression before and after the intervention (Feinstein et al., 2013).

Finally, there is little information about how the impact of yoga may differ between the four courses of MS. Also, interventions varied in duration between a few weeks to several months, but little is known about the possible impact of yoga as a long-term method of symptom management.

Future research needs to employ more thorough study designs and precise measurements. Since many studies evaluate outcomes via self-reported measures such as questionnaires and Likert scales, it is imperative to utilize study designs with suitable control groups in order to limit the effect of participant expectations on outcome. Also, it would be beneficial to use clinical imaging techniques and precise physical assessment measures to investigate if quantifiable physical changes correspond to self-reported measures of improvement.

It is unclear how the frequency, duration, or intensity yoga practice may impact symptoms experienced by patients. Future research might investigate whether a certain regimen might be more effect for improving patient outcome. Also, very few trials examining yoga and MS have conducted follow up studies. Future research should include follow up measurements to investigate if outcomes remained significant or if variables had returned to their baseline values. Furthermore, longitudinal research should be done on how yoga may impact symptom management in the different courses of MS when yoga is incorporated into a treatment plan from early stages in the disease.

### Conflict of interest statement

The authors declare that the research was conducted in the absence of any commercial or financial relationships that could be construed as a potential conflict of interest.
